# Suspected and Confirmed Acute Appendicitis During the COVID-19 Pandemic: First and Second Quarantines—a Prospective Study

**DOI:** 10.3389/fsurg.2022.896206

**Published:** 2022-06-21

**Authors:** Vidas Petrauskas, Eligijus Poskus, Raminta Luksaite – Lukste, Marius Kryzauskas, Marius Petrulionis, Kestutis Strupas, Tomas Poskus

**Affiliations:** ^1^ Clinic of Gastroenterology, Nephro-Urology and Surgery, Institute of Clinical Medicine, Faculty of Medicine, Vilnius University, Vilnius, Lithuania; ^2^ Department of Radiology, Nuclear Medicine and Medical Physics, Institute of Biomedical Sciences, Faculty of Medicine, Vilnius University, Vilnius, Lithuania

**Keywords:** acute appendicitis, complicated, COVID-19, delayed presentation, quarantine

## Abstract

**Purpose:**

COVID-19 posed an unprecedented modern global healthcare crisis affecting both elective and urgent surgeries. The aim of this study is to evaluate the difference in the presentation of acute appendicitis (AA) before and during the COVID-19 era, the first and second quarantines.

**Methods:**

We performed a prospective study from December 2018 to May 2021. Two cohorts were analysed, one with patients who presented to the emergency department (ED) with suspected AA and the second with confirmed AA. Both cohorts were divided into four groups: before COVID-19, during the first quarantine, between the first and second quarantine, and during the second quarantine. Data such as demographics, the time to first contact with the healthcare provider and time to operation, laboratory tests, clinical stage of AA, length of stay, and COVID-19 status were collected. A total of 469 patients were enrolled.

**Results:**

A total of 209 patients were male (45%) and 260 were female (55%), with the median age being 33 years (24–45). In the first cohort of suspected AA, there was no difference in sex; however, more older patients presented to the ED during the first quarantine (41 years) compared with other groups (28.5, 36, and 32.5 years), *p* < 0.000. Before the pandemic, there was a shorter duration of symptoms to first contact with the healthcare provider (13 h) compared with other groups, *p* = 0.001. In the second cohort of confirmed AA, there was a shorter period of time to operation from first symptoms before the pandemic (22 h) compared with other groups (30, 35, 30.5 h), *p* < 0.000. There were more complicated gangrenous, perforated appendicitis or periappendicular abscess in Group 2 and 3 (26, 22 and 10%, and 26, 22 and 2%, respectively) compared with Group 1 (20, 4 and 3%) and Group 4 (22, 12, and 2%), *p* = 0.009. Hospital stay was longer during the first quarantine (3 days) compared with other groups (2 days), *p* = 0.009. Six patients were COVID-19 positive: one from Group 3 and five from Group 4 (*p* > 0.05).

**Conclusions:**

Our study suggests that during the first quarantine of the COVID-19 pandemic, there was delayed presentation to the ED with suspected AA and there was a greater proportion of complicated appendicitis and longer hospitalization in confirmed cases as well.

## Introduction

A new variant of coronavirus SARS-CoV-2 (severe acute respiratory syndrome virus-2) was first detected in Wuhan, Hubei province, China, in December 2019 (1, 2). The intrinsic properties of the virus itself, its global reach, and international travel allowed a quick dissemination of the virus, and on March 11, 2020, the new coronavirus disease (COVID-19) was declared a pandemic by the World Health Organization, leading to a possibility of healthcare system collapse throughout the world (3). At the start of the pandemic, the incubation period of COVID-19 ranged from 5.2 to 12.4 days and mortality from 2 to 5%. The most vulnerable population was the elderly and patients with multiple comorbidities (4). Many countries, including Lithuania, reacted to the rapidly changing COVID-19 epidemiologic situation by declaring national quarantine and stay-at-home policies to prevent disease dissemination, healthcare system overload and mortality. However, it was possible that patients did not seek necessary healthcare due to the fear of contracting the virus in the hospital. This led to a drop in emergency department (ED) volumes by as much as 50% (5, 6). Patients presented to the ED with more advanced pathologies than usual (7).

Acute appendicitis (AA) is one of the most common surgical emergencies worldwide. A lifetime risk for AA is approximately 8.6% for males and 6.7% for females (8). It is still not clear if laparoscopic or open appendectomy is the gold standard to treat AA. However, some evidence suggests that the laparoscopic approach is feasible because of lower pain intensity on day one, less wound infections, shorter hospital stay, and time until return to normal activity in adults (9, 10). AA can lead to a rapid clinical deterioration, with perforation rates of 16% to 40%, especially in children and in patients over 50 years of age (11). The main risk factor of complicated appendicitis is delayed admission to hospital (12, 13).

We conducted a prospective study to evaluate what kind of impact did the COVID-19 pandemic and the first and second quarantines have on the time to presentation and rate of complications of AA.

## Patients and Methods

The study was approved by the Institutional Review Board and Regional Committee of Bioethics. Informed consent was obtained from all patients for participation in the study.

A total of 469 patients with suspected AA in the ED were included from December, 2018 to May, 2021. Two cohorts were analysed: the first cohort included all patients with suspected AA and the second included confirmed AA clinically and radiologically (US or CT, or both).

Four groups were created by the date of presentation to the ED. Group 1 included patients from the prepandemic period (December, 2018 to March 3 2020), Group 2— first quarantine (March 3, 2020 to June 17, 2020), Group 3—interquarantine period (June 17, 2020 to November 7, 2020), and Group 4—second quarantine (November 7, 2020 to May, 2021).

The radiologic features of AA were defined by wall thickening (>2 mm), appendiceal dilatation (>6 mm in diameter), and additional features such as periappendicular fat inflammation or periappendicular fluid collection and lymphadenopathy. Patients in the complicated AA group were those with radiologic evidence of perforated appendix, with a periappendicular abscess or gangrenous AA. If diagnosed intraoperatively, perforated appendicitis was visualized by the surgeon and recorded in the operative protocol. All patients with radiologically confirmed AA were operated upon, and none was treated with antibiotics alone. All the removed appendices were evaluated by a pathologist.

The inclusion criteria were as follows: patients older than 18 years, those who presented in the ED with suspected AA, and those who wrote an informed consent for participation in the study. The exclusion criteria were as follows: patients under 18 years of age and those who refused to participate in the study.

We collected data including demographics (age, sex), the time from the onset of symptoms to first contact with the healthcare provider, the time from first symptoms to operation, laboratory tests (white blood cell count, neutrophil count, and C-reactive protein), length of hospitalization, COVID-19 status, and the final results, including the pathology report (diagnostic laparoscopy, uncomplicated appendicitis, gangrenous appendicitis, perforation, periappendicular abscess and other pathologies).

The primary endpoint was the time from first symptoms to contact with the healthcare provider in the ED. The second endpoint was the rate of complicated AA including gangrene, perforation or periappendicular abscess.

### Statistical Analysis

Continuous variables were expressed as mean and standard deviation or median with interquartile range. Categorical variables were reported as counts and percentages. Statistical significance was determined by using the 2-tailed Kruskal–Wallis test for continuous variables with no Gaussian distribution or the *χ*^2^ test for categorical variables. All statistical analyses were performed using SPSS (SPSS 21.0; Windows version, SPSS Inc, Chicago IL, USA). A p-value <.05 was considered statistically significant.

## Results

**Figure 1:**
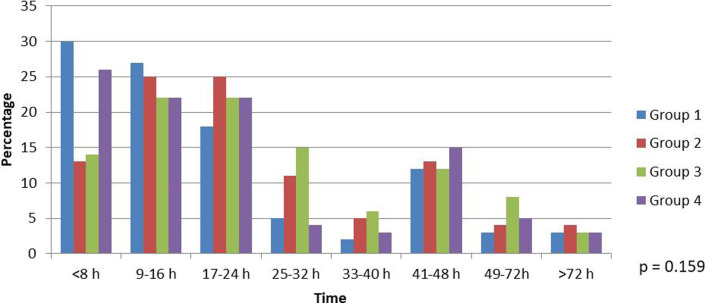
Time from onset of symptoms to first contact with healthcare provider in suspected acute appendicitis cohort.

A total of 209 male and 260 female patients with a median age of 33 (interquartile range 21; 24–45) participated in the study. The patients’ characteristics of the total cohort of suspected AA (*n* = 469) are given in Table 1. Younger patients presented to the ED before the COVID-19 pandemic (28.5 years) compared with other groups (41, 36 and 32.5 years, respectively). There was no difference in sex between the groups. The patients presented to the ED with suspected appendicitis up to 10 h earlier before the pandemic (13 h) compared with the first quarantine (22 h) and interquarantine (24 h) periods. During the second quarantine, patients received medical help similar to that during the pre-COVID period (Table 1 and Figure 1).

**Table 1:** Cohort of suspected acute appendicitis.

	All patients (*n* = 469)	Group 1 (*n* = 202)	Group 2 (*n* = 55)	Group 3 (*n* = 64)	Group 4 (*n* = 148)	*p*-value
Age	33 (21; 24–45)	**28.5 (18; 22–41)**	41 (24; 32–56)	36 (25; 26–50)	32**.**5 (19; 25–43)	**0**.**000**
Sex						0.681
Male	209 (45)	85 (42)	23 (42)	30 (47)	71 (48)	
Female	260 (55)	117 (58)	32 (58)	34 (53)	77 (52)	
Time from symptoms to the healthcare provider	17 (21; 9–30)	**13 (19; 6**–**26)**	22 (24; 10–34)	24 (28; 12–39)	17**.**5 (24; 8–32)	**0**.**001**
WBC	11**.**8 (6; 8–14)	11**.**5 (6; 8–14)	11**.**8 (4; 11–14)	12**.**2 (6; 10–16)	11**.**9 (6; 8–14)	0.138
NEU	9**.**4 (6; 6–12)	8**.**8 (6; 6–12)	9**.**8 (4; 8–12)	9**.**5 (5; 8–13)	9**.**6 (6; 6–12)	**0**.**034**
CRP	14**.**2 (44; 2–46)	7**.**6 (23; 1–24)	28**.**9 (100; 7–107)	34**.**7 (86; 8–94)	14.4 (48; 3–51)	**0**.**000**

*WBC, white blood cell count; NEU, neutrophil count; CRP, C reactive protein count. P value <0.05 considered statistically significant. Continuous variables in median and interquartile ranges.*

*Meaning of bold in the p value column is for statistical significance. In other parts of the table – a notable finding.*

The characteristics of patients with confirmed AA and those operated upon (*n* = 325) include additional information on time to operation, laboratory tests on admission, length of stay, and COVID-19 status (Table 2). Similar to the suspected AA cohort, older patients presented to the ED during the first quarantine and interquarantine periods (*p* = 0.002), and the time from the onset of symptoms to first contact with the healthcare provider was also longer in these groups (*p* = 0.006). Before the COVID-19 pandemic, patients were operated upon for AA earlier (22 h from the onset of symptoms) compared with the COVID-19 period - (30–35 h), *p* = 0.000. White blood cell count and neutrophil count were similar between the groups; however, the CRP level was higher in Groups 2 and 3 (33.7 and 44.5 mg/L) compared with that in Groups 1 and 4 (13.6 and 22.6 mg/L), *p* = 0.000. Hospitalization was longer during the first quarantine (3 days vs 2 days in other groups), *p* = 0.009. One patient tested SARS-CoV-2 positive from Group 3 and five patients tested SARS-CoV-2 positive in Group 4; none of the patients required oxygen supplement and were not experiencing dyspnea. Two patients with detected virus were operated with open appendectomy, and the other four were operated with laparoscopic appendectomy.

**Table 2:** Cohort of confirmed acute appendicitis.

	All patients (*n* = 325)	Group 1 (*n* = 115)	Group 2 (*n* = 50)	Group 3 (*n* = 50)	Group 4 (*n* = 110)	*p*-value
Age	35 (21; 27–48)	33 (20; 25.5–45.5)	**42 (24; 32–56)**	**41 (23; 30–53)**	34 (19; 26–45)	**0**.**002**
Sex						0.520
Male	171 (52.6)	65 (56.5)	22 (44)	27 (54)	57 (51.8)	
Female	154 (47.4)	50 (43.5)	28 (56)	23 (46)	53 (48.2)	
time from symptoms to the healthcare provider	18 (21; 9–30)	13 (17; 7–24)	**21.5 (22; 1032)**	**24 (24; 12**–**36)**	18 (27; 9–36)	**0**.**006**
time from symptoms to operation	28 (23; 20–43)	**22 (18; 16**–**34)**	30 (18; 25–43)	35 (22; 24–46)	30.5 (27; 21–48)	**0**.**000**
WBC	12.7 (5.4; 10.2–15.6)	13.2 (5; 10.4–15.4)	12.1 (4.3; 10.6–14.9)	12.5 (5.8; 10.1–15.9)	12.6 (6.6; 8.9–15.5)	0.754
NEU	10.5 (5; 8–13)	10.6 (4.6; 8.2–12.8)	10.2 (4; 8.6–12.6)	10 (5.4; 8.1–13.5)	10.6 (6.4; 6.7–13.1)	0.953
CRP	19.6 (59; 6.5–65.5)	13.6 (38. 1;4.1–42.2)	**33.7 (99.1; 8.2**–**107.3)**	**44.5 (77.7; 16.8**–**93.5)**	22.6 (63; 7.8–70.8)	**0.000**
Length of stay	2 (2; 2–4)	2 (1; 2–3)	**3 (3; 2**–**5)**	2 (2; 2–4)	2 (3; 1–4)	**0**.**009**
COVID–19						0.057
Positive	6 (1.8)	0 (0)	0 (0)	1 (2)	5 (4.5)	
Negative	319 (98.2)	115 (100)	50 (100)	49 (98)	105 (95.5)	

*WBC, white blood cell count; NEU, neutrophil count; CRP, C reactive protein count. P value <0.05 considered statistically significant. Continuous variables in median and interquartile ranges.*

*Meaning of bold in the p value column is for statistical significance. In other parts of the table – a notable finding.*

**Figure 2:**
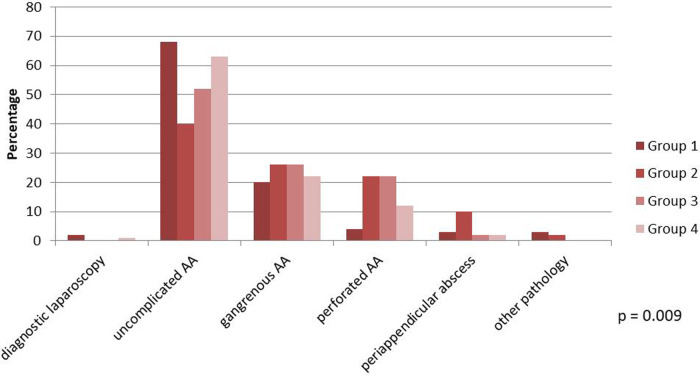
Results of patients operated for acute appendicitis.

The final diagnosis for those operated for AA showed that a significant proportion of patients during the first quarantine (Group 2) presented with more advanced disease, with 22% of the patients presenting with perforation and 10% of them presenting with periappendicular abscess (Figure 2).

## Discussion

Because COVID-19 was declared a global pandemic, it was expected that a high influx of patients with respiratory symptoms would increase the burden on hospitals, especially the intensive care units (14). Reacting to the rapidly evolving situation related to COVID-19, several surgical guidelines with recommended changes in delivery of surgical care were released, including those by the Royal College of Surgeons and the American College of Surgeons (15, 16). Briefly, the recommendations included a postponement of elective operations as well as consideration of non-operative management with antibiotics for uncomplicated AA as the initial treatment of choice. The rationale for this decision was previous studies showing that non-operative management in uncomplicated appendicitis was safe in the short term (17–20). Also, a high risk of pulmonary complications (51%) and mortality (up to 24%) was reported for those with positive SARS-CoV-2 and undergoing surgery (21). This strategy was adopted by UK surgeons, and short-term results were promising (22, 23). Another issue was the possibility of the spread of the virus with surgical smoke plumes generated during laparoscopy, and, therefore, guidelines suggested performing appendectomy through laparotomy (24). In our study, 2 patients with positive SARS-CoV-2 were operated with open appendectomy and 4 with the laparoscopic approach. Laparoscopic appendectomy was chosen by our institution because, since January 2021, most of our personnel had been vaccinated and using personal protective equipment. The Society of American Gastrointestinal and Endoscopic Surgeons guidelines of 2022 suggest that the decision of performing laparoscopic surgery instead of an open one for COVID-19 positive patients should be based on individual patient characteristics (25). However, more evidence is necessary to prove that the spread of SARS-CoV-2 with surgical plumes is harmless.

A majority of the patients (98.2%) in our study were treated with laparoscopic appendectomy with the conversion to laparotomy only if necessary. Periappendicular abscesses were not identified radiologically before the operation and did not exceed 2 cm in diameter once opened, and, therefore, no patient was treated with drainage and antibiotic therapy alone.

Negative appendectomy rates in the literature rise up to 20% (26). High numbers are seen in the UK, where it is still common to diagnose AA clinically (26). In our study, there were no negative appendectomies. If the surgeon suspected a normal appendix at the beginning of the operation and no other pathology was observed, the intervention was completed as a diagnostic laparoscopy. Another reason for this is our institution’s policy of the liberal use of CT if the diagnosis is not clear after US. MRI is used for pregnant patients after equivocal US (27–29).

### Time to Contact With the Healthcare Provider

It was observed early during the first quarantine that not only fewer patients admitted themselves to the ED, but also those who sought medical help presented with advanced disease (30). *Gao* et al. from China reported that the time from the first symptoms of AA to hospitalization increased from 17.3 h in pre-COVID to 65 h during the pandemic (9). *Tankel* with colleagues in Jerusalem observed the same phenomenon—delayed presentation (>3 days) increased from 2.8% to 5.9% (30). Another study from Argentina by *Angeramo* et al. reported a delay in consultation from 34 h before the COVID-19 pandemic to 54 h, *p* < 0.000. We observed similar results in our study. Before the first quarantine, patients contacted the healthcare provider in 13 h compared with 21.5 h when the pandemic broke out (*p*  = 0.006).

### Complicated Acute Appendicitis

It was observed worldwide that during the first quarantine, when new restrictions were implemented by governments, there was a trend toward conservative treatment of AA at home first, and more patients presented to the ED only when complications occurred. Many authors reported a higher proportion of gangrenous, perforated appendicitis or periappendicular abscess. In a cohort of 378 patients, *Tankel et al.* observed an increase of complicated appendicitis from 13.1% in the pre-COVID era to 20.6% during the pandemic (30). *Finkelstein et al.* from the USA reported 33% of complicated appendicitis compared with the previous year of 17%, *p* = 0.04. A retrospective study of *Angeramo* with colleagues from Argentina also observed more advanced disease in COVID-19 times, with complications occurring twice as common (38%) compared with the pre-COVID19 era (19%) (31). An even higher increase in the proportion of gangrenous, perforated appendicitis or periappendicular abscess was reported in China by *Gao* et al. (12.4 vs 51.7%) and the USA by *Orthopoulos* et al. (16.7 vs 73.5%) (9, 32). Our study results were no different. Before the pandemic, 27% of patients presented with complicated appendicitis compared with 58% during the first quarantine, 50% in the interquarantine period, and 36% during the second quarantine, *p* = 0.009.

With more cases of complicated AA, longer hospitalization and increased costs are inevitable. In our study, length of stay was longer during the first quarantine (3 days) compared with that during other periods (2 days), *p* = 0.009. This is an additional burden to the healthcare system, especially during these challenging COVID-19 times.

Complicated AA has some serious long-term consequences. These include mechanical ileus due to intra-abdominal adhesions (1%) and possible bowel resections afterwards, incisional hernia (0.7%) and ectopic pregnancy (OR 1.78 95% CI 1.46–2.16, *p* < 0.0001) (33, 34). All effort must be directed towards the timing of appendectomy before perforation or abscess formation.

To our knowledge, this study is the first perspective observational study to analyse time from the onset of symptoms to contact with the healthcare provider and the rate of complications of AA during the first and second quarantine periods of COVID-19 in Lithuania.

Our study has some limitations as well. It was conducted in a single tertiary centre. No randomization or blindness was possible because of the nature of the study. Also, we had only a small sample size. Patients were assigned to one of four groups because of the time of presentation to the ED, and they were not case-matched by comorbidities or BMI.

## Conclusions

The COVID-19 pandemic and the restrictions that have followed pose a real challenge to modern healthcare systems. Because of the fear of contracting the virus, patients with AA sought medical help later than usual. As a consequence, the patients presented with more advanced stages of AA including gangrene, perforation or periappendicular abscess during the first quarantine. Prolonged hospitalization in complicated cases is inevitable. In the second quarantine, however, this phenomenon was no longer observed.

## Data Availability

The raw data supporting the conclusions of this article will be made available by the authors, without undue reservation.
